# Empowering women through probiotic fermented food in East Africa

**DOI:** 10.7189/jogh.10.010330

**Published:** 2020-06

**Authors:** Gregor Reid, Wilbert Sybesma, William Matovu, Arnold Onyango, Nieke Westerik, Remco Kort

**Affiliations:** 1 Lawson Health Research Institute, London, Ontario, Canada; 2 Departments of Microbiology & Immunology and Surgery, Western University, London, Ontario, Canada; 3 Yoba for Life Foundation, Amsterdam, the Netherlands; 4 Heifer International, Kampala, Uganda; 5 Jomo Kenyatta University of Agriculture and Technology, Kenya; 6 VU University Amsterdam, the Netherlands; 7 ARTIS-Micropia, Amsterdam, the Netherlands

The role of women in society has evolved in the developed world, but in many developing countries it still revolves around food production and preparation and caring for children in a gender inequitable environment. Many females are exposed to violence, and in poverty-stricken areas with rampant malnutrition, women’s dependency on males for shelter and access to farmland can restrict their personal growth [1].

Confounding factors include lack of education, societal traditions that favour males, malnutrition and insufficient local food production, unemployment especially amongst youth, poor transportation, high rates of infant mortality, and poor access to clean water, electricity, housing and heat. Women in such impoverished settings find it difficult to become empowered and able to establish an independent means of generating revenue. No single intervention can solve all these issues, but we would propose that utilization of beneficial microorganisms (probiotics) can make a significant impact.

## LESSONS FROM HISTORY

Humans have long utilized bacteria and yeast to produce fermented foods, and as a result benefited in many ways health-wise [2]. But, the globalization of food and need for more to service the growing population has resulted in the wider use of chemical pesticides, herbicides and fertilizers, and a departure from traditions of fermenting foods such as millet, to consumption of maize, sugary drinks and processed foods [3]. The net effect is elevated rates of obesity, metabolic syndrome and cardiovascular and chronic respiratory and allergic diseases. Illness and death from children consuming corn and peanuts contaminated by aflatoxins is a stark example of a negative outcome of poor food practices [4].

## A NEW APPROACH CENTRED ON FERMENTED FOOD AND EMPOWERING WOMEN

Many decades of research and trials in the field of nutrition sciences yielded numerous feeding ideas for interventions, but few of them led to societal change. This may reflect general concerns about the field of nutrition sciences, in that it relies too much on small short-term interventions with little relevance to the real world [5].

**Figure:**
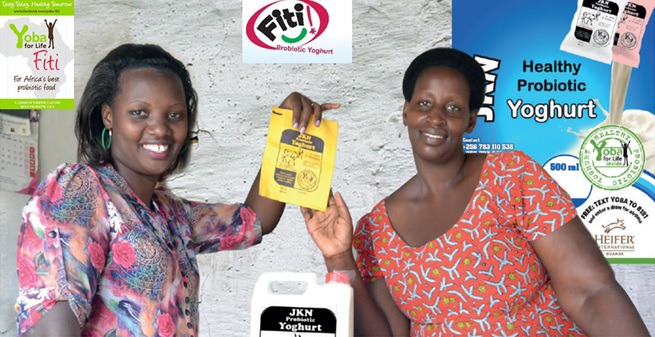
Photo: From the collection of Heifer International (used with permission).

We hypothesized that societal change could occur if women received training on the production of probiotic fermented food as a means to allow them to generate income and financial independence, and provide nutritious, health-promoting food to their family and community. This approach was based upon the well-documented attributes of two probiotic *Lactobacillus rhamnosus* strains [6,7], plus a *Streptococcus thermophilus* starter culture combined into a 1 g sachet that allows production of 100 L of probiotic fermented food.

The approach was based upon empowering women as the anchors of the community and providing them with the means to utilize local resources – milk, fruit, millet and other cereals. Through a pro-poor business model that increased household income, women stepped forward as home owners with their own dairy cows and thereby increased income sufficiently to send their children to better schools. The profits increased by volumes sold, especially over 50 L per batch.

A book summarizing case studies illustrates the profits made, poverty eradication, businesses established, gender equality and impact on schools and society [8]. By generating more revenue, a woman can afford to send the children to better schools or access medical facilities. She can purchase a motor cycle or car to deliver products but also allow access to larger hospitals and clinics distant to the rural residence. It can provide funds to move from a mud house to a concrete home that is more secure, better insulated and located in an area that may be less susceptible to theft and violence.

This model is most certainly transferable. If women in any developing country have access to a food source like milk, cereal or fruit, they can with only limited hardware and an energy source, produce and sell these fermented foods. As an example, the model has already been transferred to Nepal and Senegal.

**Figure 1:**
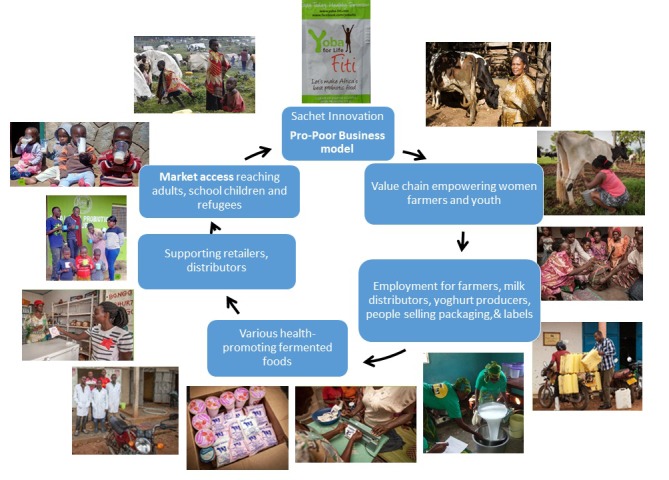
The value chain impacted by the sachet technology enabling probiotic fermented food production in East Africa.

The educational program covering all aspects of the production process (hygiene, distribution, sales, marketing, book-keeping) encouraged participation at the African sites. The net effect was appreciating the taste and texture of the fermented foods, and the creation of a value chain that brought income to many people (**Figure 1**).

This pro-poor model has improved food and nutrition security by increasing local production, distribution, and consumption of probiotic fermented foods – not for a particular time window, but in a sustainable manner. This was possible even in a country such as Uganda where in 2015 25% of the 35 million population was defined as poor [9]. By providing access to sachets through trainers and dairy networks, the geographical challenge of covering 1 263 310 square miles in Uganda, Kenya and Tanzania became feasible, with so far best coverage in Uganda. The resultant 262 production units reaching 260 000 weekly consumers in the three countries within two years shows the potential for this concept.

No such pro-poor model exists anywhere that provides a means to produce probiotic fermented food at the grass roots level. People in developed countries can purchase yoghurt-making systems and organisms but these are designed for family use at a cost much more than the African units. In addition, people have made small batch yoghurt for centuries, such as Dahi in India [10], but not with such a probiotic formulation. Indeed, this supplementation with a probiotic strain has been shown to be superior to Dahi in treatment of diarrhea in children [10].

Such is the feasibility of the model, we applied to set-up a PU unit in London Ontario with sales targeted to low income and homeless members of society. This was achieved but the health authorities demanded that the yogurt be produced by a commercial manufacturing plant, quadrupling the retail price to unaffordable levels for poor people, and failing to mimic the local production performed by the African sites.

While hygiene and food safety are understandably priorities in developed countries, the acidification process of fermentation kills most pathogens and contamination can be easily monitored. We therefore recommend more effort be made to establish production units catering for impoverished and malnourished residents of developed countries.

## SETTING REALISTIC GOALS

While the establishment of goals is an essential component of any research project, and a requirement of the funding agency, this cannot be done by isolating the beneficiaries. They must be allowed to convey their needs and indicate how they will contribute. Success can be noted when the ‘western’ trainers are replaced by locals who have received training and now impart lessons to others in the community. Engagement of inspectors from the Dairy Development Authority further strengthen the credibility of the PUs.

Communication must be sensitive to language and dialect, how people acquire news and react to it. Television, radio, roadshows, community fairs, and a full-length feature film, *The Promised Land* depicting the food production process were all utilized.

As school feeding programs are common in these countries, a number of the producers focused their efforts on providing fermented foods to the schools. Several studies have been carried out at these schools with results showing reduced skin allergies from consuming the probiotic yoghurt (Westerik et al. unpublished). This school outreach provides a means through which millions of lives could be impacted. By linking parents and schools to PUs, steps are under way to expand the reach of these health-promoting foods and in some cases replace regular cereal porridge or milk. With maize and millet sold in markets and local households being often contaminated by aflatoxins and coliforms, the provision of fermented probiotic cereal free of these substances, further strengthens the desire for schools to participate.

## KEYS TO SUCCESS

Identifying local champions and change-makers is an important factor for success. For example, the leader of a women’s group Mekono Yetu in Tanzania, Ms. Maimuna Kanyamala, is highly respected and well-connected in the country. Her efforts boosted the number of PUs from 10 to over 80 in Tanzania and connected producers to policy makers and various levels of government. One woman, Winnie Busingye established the Kiboga Ikamiro Women’s Group PU in Uganda and attended a conference in Ottawa in 2018 to share her experiences. The sachets and pro-poor model enabled her to transition from poverty and a thatched single room dwelling to owning 20 dairy cows, employing 27 staff, sending her four children to better schools, owning a new concrete home with iron roof, and producing up to 200 L of probiotic yogurt per day. While this does not mean Winnie has lowered her risk all diseases per se, she now has the means to access medical treatment and nutritious food, and the financial independence that can prevent women having to trade sex for food, as many counterparts have done resulting in the spread of HIV [11].

The empowerment of women is illustrated by gender governance, with seventy per cent ownership of PUs across the three countries. This is a significant change from previous roles of making food only for the family or selling someone else’s product by the roadside.

A study of franchise restaurants in Kenya showed that brand power/concept, cultural appeal, excellent choice of franchisees, good relationship with franchisees, site selection or location, and distance management are important for success [12]. Thus, people with experience in microenterprises, and particularly in dairy business, can lead to better uptake and fewer drop-offs.

## IN SUMMARY

The application of probiotic fermented food has the potential to reach millions of people. For example, twenty Early Childhood Development Centres in Kenya now use the sachets to produce fermented maize-millet-soybean porridge; and there are fifty thousand of these serving three million children.

This project has shown how ‘western’ science and local ingenuity can impact vulnerable populations in rural East Africa. It has the potential to improve a number of important health and economic markers in regions challenged by poverty, infectious diseases, malnutrition and unemployment.

As yet, the introduction of such a microbiome food-based initiative has not been assessed to see if and how it effects disease prevalence, morbidity and mortality. The premise exists for it to do so, especially through the women who have risen to the challenge of producing these foods, and those along the value chain who have witnessed greater revenue generation without denying the poorest members of society access to the products. Others agree that gendered empowerment can help prevent HIV spread [13]. By doing this through a practical, affordable and community-wide initiative using different food sources, we believe that this model can be transferable across the globe.
